# Comparison of a rapid visual algorithm for quantification of infarct size with direct planimetry of infarct size by delayed enhancement-CMR

**DOI:** 10.1186/1532-429X-11-S1-O22

**Published:** 2009-01-28

**Authors:** Omar M Cheema, Ankit A Patel, Dipan J Shah

**Affiliations:** Methodist DeBakey Heart & Vascular Center, Houston, TX USA

**Keywords:** Infarct Size, Leave Ventricular Mass, Bland Altman Analysis, Myocardial Infarct Size, Visual Algorithm

## Introduction

Direct planimetry of myocardial infarct size by delayed enhancement CMR (DE-CMR) has been well validated as a technique for quantification of infarct size with both a high degree of accuracy and reproducibility. Direct planimetry however requires extensive post processing and is time consuming and therefore not ideal for performance in a routine clinical service. We propose a visual algorithm to quantify total infarct size that would be rapid and easily incorporated into routine clinical practice. In this study we sought to: 1) compare the level of agreement between our visual scoring algorithm and direct planimetry of infarct size; 2) compare the time required for each method of quantifying infarct size.

## Methods

We enrolled 101 consecutive patients with known chronic myocardial infarction (MI) who underwent CMR viability assessment. Direct planimetry was performed by segmentation of hyperenhanced (HE) regions (signal intensity > 2 standard deviations above remote myocardium) in all short axis DE-CMR slices. The total volume of the HE zone was then divided by the total volume of myocardium within the left ventricle (calculated by planimetry of endocardial and epicardial borders on all DE-CMR images so as to include both HE and non-HE myocardium). Visual scoring of all studies was performed using a 17 segment model with scores assigned based on the visual extent of HE myocardium in each segment (i.e. 0 = no HE, 1 = 1–25% HE, 2 = 26–50% HE, 3 = 51–75% HE, and 4 = 76–100% HE). Visual infarct size was then calculated by averaging the midpoint of the % HE within each of the 17 segments (i.e. score of 0 = 0%, 1 = 13%, 2 = 38%, 3 = 63%, 4 = 88%). Agreement between the visual algorithm and planimetry was compared using Bland-Altman analysis.

## Results

The study population consisted of 101 patients with chronic MI (infarct age 5.3 ± 6.5 years) of which 70 (69%) were men, age 63.7 ± 2.3 years, left ventricular (LV) ejection fraction 52.4 ± 15.5%, and LV mass 154.7 ± 51.3 g. Of the 101 patients, 30 (30%) had a history of congestive heart failure, 29 (29%) had diabetes mellitus, 86 (85%) had hypertension, and 94 (93%) had hyperlipidemia. The total infarct size was not statistically different between the visual algorithm (11.4 ± 10.4%) and directly planimetry (9.3 ± 8.5%, p = NS). The range of infarct sizes was 0–40% by direct planimetry. Bland Altman analysis revealed a nonsignificant bias of 2.1% of LV (p = NS) with 95% confidence interval between the visual algorithm and planimetry of +11.3% to -7.1%, see figure. The visual algorithm required less time than direct planimetry (5.5 ± 1.6 minutes vs. 22.4 ± 4.5 minutes respectively; p < 0.001).

## Conclusion

The visual algorithm provides a rapid method to quantify total infarct size with good agreement to direct planimetry. This is a useful time-efficient alternative for quantifying chronic infarct size in a routine clinical setting.

